# Skin Melanin Content and the Foveal Avascular Zone Correlation in a Healthy White Population

**DOI:** 10.1167/iovs.67.3.43

**Published:** 2026-03-18

**Authors:** Valentina Folegani, Mariano Cozzi, Andrea Menghi, Salvatore Parrulli, Francesco Romano, Alessandro Invernizzi, Giovanni Staurenghi

**Affiliations:** 1 Eye Clinic Luigi Sacco Hospital, Department of Biomedical and Clinical Sciences, University of Milan, Milan, Italy; 2 Department of Ophthalmology, University Hospital Zurich and University of Zurich, Zurich, Switzerland; 3 Save Sight Institute, Discipline of Ophthalmology, Sydney Medical School, The University of Sydney, Sydney, New South Wales, Australia

**Keywords:** foveal avascular zone, melanin, optical coherence tomography angiography, optical coherence tomography, white, caucasian

## Abstract

**Purpose:**

This study aimed to investigate the correlation between skin melanin content and foveal avascular zone (FAZ) characteristics in healthy White subjects and to determine whether melanin expression influences foveal morphology.

**Methods:**

In this cross-sectional study, 154 eyes from healthy volunteers 18 to 55 years old underwent comprehensive ophthalmic examinations including optical coherence tomography angiography (OCTA) imaging. The FAZ area was manually delineated from OCTA images. Skin melanin content was measured using a colorimeter at three anatomical sites to obtain a melanin index. Pearson’s correlation and multivariable linear regression analyses were performed to assess associations among the melanin index, FAZ area, axial length (AL), central foveal thickness (CFT), and the presence of persistent foveal inner retinal layers.

**Results:**

A statistically significant positive correlation was observed between the melanin index and FAZ area (*r* = 0.394, *P* < 0.001). Multivariable analysis revealed that each 100-unit increase in the melanin index was associated with a 0.12-mm^2^ increase in FAZ area (*P* < 0.001), whereas age, sex, and AL were not found to be associated with FAZ. Furthermore, a lower melanin index was significantly associated with the likelihood of foveal inner retinal layer persistence (odds ratio = 0.98) and higher CFT (β = −0.11, *P* = 0.02).

**Conclusions:**

These findings suggest that physiological variations in cutaneous melanin may be associated with FAZ dimensions and foveal architecture in healthy White individuals. Melanin-related pathways appear to play a regulatory role in retinal development, highlighting the need to consider pigmentation when using FAZ measurements as biomarkers in ocular and systemic diseases.

The foveal avascular zone (FAZ) is the capillary-free central region of the fovea, densely populated by cone photoreceptors with elongated outer segments, and it plays a critical role in high-acuity vision.^1^ The advent of optical coherence tomography angiography (OCTA) has significantly advanced our ability to non-invasively image the retinal vasculature, including detailed visualization of the FAZ.^2^ Previous studies have consistently shown that the FAZ area is smaller in the superficial vascular plexus (SVP) compared to the deep vascular plexus (DVP), likely due to anatomical differences in these retinal layers.^3^^–^^5^ Despite this, measurements in the two plexuses remain highly correlated in both shape and size.^2^ Considerable interindividual variation in FAZ size and morphology has been observed in healthy eyes,^6^^,^^7^ reflecting its developmental significance. FAZ formation precedes and likely facilitates excavation of the foveal pit, underscoring its critical role in normal foveal development.^8^^,^^9^ The human foveal depression begins to emerge by late gestation with the establishment of a nascent FAZ and continues to enlarge postnatally, driven by anti-proliferative and/or anti-angiogenic factors.^10^

In recent years, the FAZ has gained increasing interest due to its association with both maturation during childhood and aging in adulthood^11^ and its high sensitivity to retinal ischemic insults. In diabetic retinopathy, for example, FAZ enlargement reflects progressive capillary dropout and correlates with disease severity and progression.^12^^–^^15^ Conversely, smaller or absent FAZ areas have been reported in conditions such as prematurity^16^^,^^17^ and albinism.^18^^,^^19^ In albino patients, disrupted melanin biosynthesis, particularly the deficiency of levodopa (l-DOPA) production from tyrosine within retinal pigment epithelium (RPE) melanosomes, appears to play a pivotal role in FAZ underdevelopment. l-DOPA acts as a ligand for G protein-coupled receptor 143 (GPR143), and its activation promotes the expression of pigment epithelium-derived factor (PEDF), a potent anti-angiogenic molecule.^20^^–^^22^ In albino retinas, reduced PEDF expression is believed to impair FAZ formation and contribute to foveal hypoplasia.^23^

Because the impact of melanin deficiency on absent or underdeveloped FAZ and on foveal hypoplasia in albinism is well documented, we hypothesize that melanin may also play a critical role in foveal, as well as in FAZ morphogenesis under physiological conditions.^24^^,^^25^ Specifically, it is plausible that interindividual differences in melanin levels could contribute to variability in FAZ size and foveal morphology. Therefore, the aim of our study was to investigate whether variable expression of skin melanin among healthy White participants is associated with FAZ characteristics and, consequently, foveal morphology.

## Methods

This observational, cross-sectional study was conducted at the Eye Clinic of Luigi Sacco Hospital (University of Milan, Milan, Italy) between September and December 2024. The study adhered to the tenets of the Declaration of Helsinki and received approval from the local Ethics Committee (approval no. 19.22). All participants were required to sign a written informed consent prior to enrollment.

### Study Population

Healthy White subjects 18 to 55 years old were recruited from among volunteer students and healthcare professionals affiliated with the Department of Clinical and Biomedical Sciences (University of Milan) and Luigi Sacco Hospital. The maximum age of 55 years was set to minimize the risk of ocular alterations such as macular disease or media opacities. Self-reported sex at birth (male/female) was recorded at enrollment as part of the demographic data collection; gender identity was not separately assessed. In addition, to be included in the study, the following criteria had to be fulfilled: best-corrected visual acuity (BCVA) higher than 80 Early Treatment for Diabetic Retinopathy Study letters (equivalent to Snellen 20/25 or better), stable central fixation, clear ocular media, and no self-reported history of ocular or systemic disease. Exclusion criteria included history of prematurity (defined as birth before 37 weeks of gestational age), refractive error with spherical equivalent greater than −6 and +3 diopters, and any prior ocular surgery, retinal alterations, or use of medications that could potentially affect the retina.

### Protocol

Each subject underwent a comprehensive ophthalmic examination including BCVA measurement, slit-lamp biomicroscopy, and dilated fundus examination. In addition, the following imaging exams were performed on both eyes of each subject: (1) axial length (AL) measurement with anterior segment OCT (ANTERION; Heidelberg Engineering, Heidelberg, Germany); (2) imaging with n investigational high-resolution spectral-domain macular OCT system (High-Res OCT; Heidelberg Engineering) with 20° × 20° volume scans comprised of 49 horizontal B-scans (resolution, 1024 × 48 pixels), averaging 16 frames per B-scan; and (3) macular OCTA with the Heidelberg SPECTRALIS OCT2 (10° × 10° scan angle, 5.7-µm/pixel lateral resolution), capturing a retinal section of 2.9 × 2.9 mm for SVP analysis (Heidelberg Engineering). To account for potential variability in retinal image magnification, individual corneal curvature values were incorporated into the imaging system calibration, as previously described by Delori et al.^26^ This adjustment ensured that FAZ area and related morphometric measurements were not biased by inter-individual optical differences.^27^ All of the examinations were performed in the morning to reduce time-of-day variability.^28^

### Imaging Analysis

**Figure 1.**
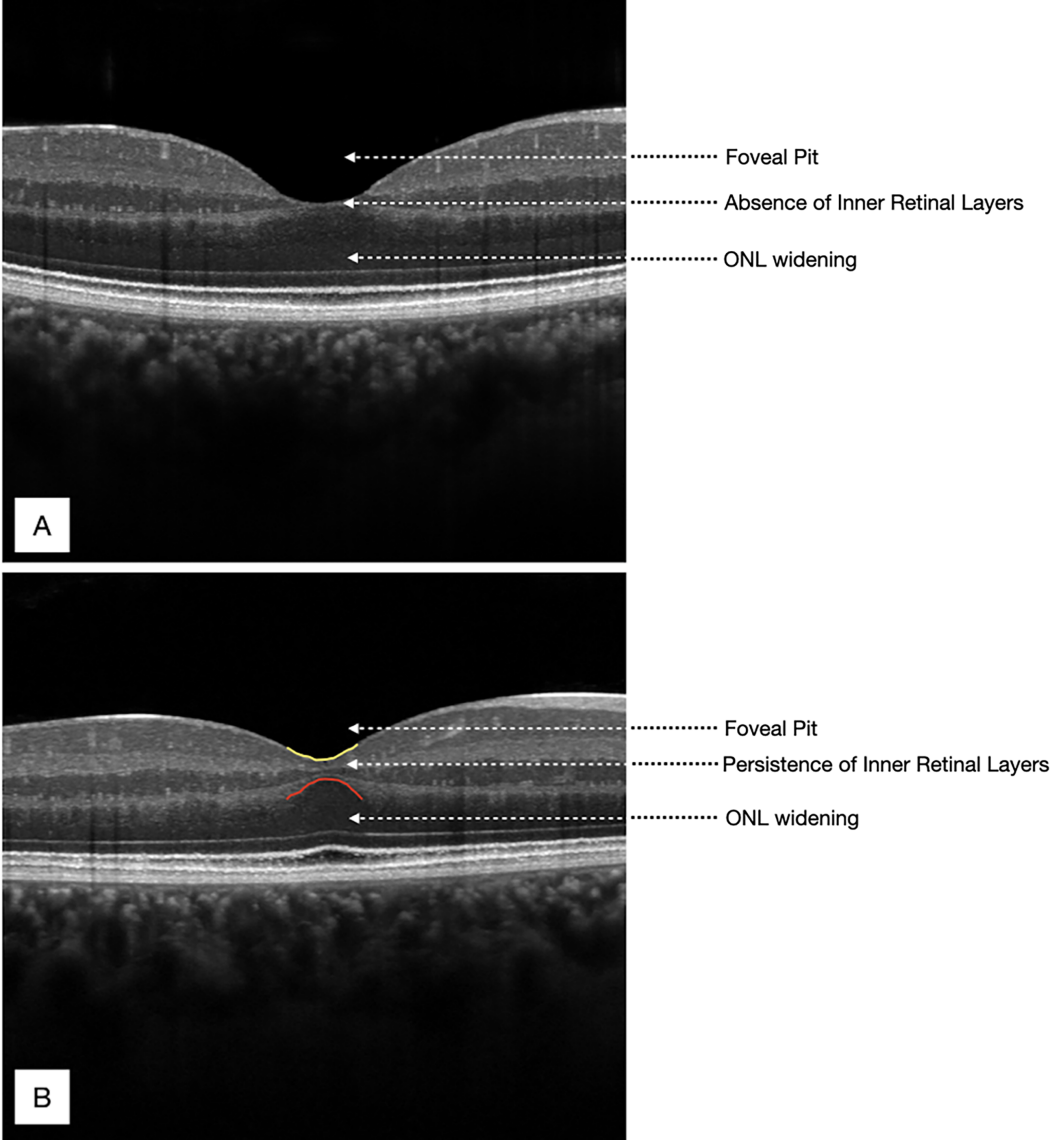
Two foveas with different structures revealed by OCT. (**A**) A normal fovea with complete extrusion of the foveal inner retinal layers and a fully formed and well-defined foveal pit. (**B**) Mild foveal hypoplasia, characterized by incomplete extrusion of inner retinal layers and a shallower foveal pit.

A single eye per subject was randomly selected for analysis, given the established interocular symmetry in FAZ and foveal morphology in healthy individuals.^26^ Each macular OCT volume was manually reviewed to determine the B-scan image with the deepest point of the foveal pit corresponding to the location with the minimum internal limiting membrane (ILM)-to-RPE (retinal) thickness on the foveal profile. This scan was used to assess central foveal thickness (CFT), measured from the ILM to the bottom of the RPE–Bruch's membrane complex using the automated segmentation provided by HEYEX 6.16.2 (Heidelberg Engineering). Central macular thickness (CMT) was also automatically calculated at the central subfield (1-mm diameter) of the macular region. In addition, mild foveal hypoplasia was assessed qualitatively, defined by the persistence of the foveal inner retinal layers above the outer nuclear layer (ONL) (Fig. 1) consistent with the Leicester Grading System for Foveal Hypoplasia^29^ and prior OCT descriptions of the foveal hypoplasia spectrum.^30^

En face OCTA images were imported into ImageJ 1.54p (National Institutes of Health, Bethesda, MD, USA).^31^ A customized slab was applied to each volume to enhance the visualization of the superficial FAZ. Specifically, a dedicated slab extending from the ILM to the inner surface of the inner plexiform layer (IPL) was manually adopted to isolate the superficial vascular plexus. OCTA scans in which the FAZ could not be clearly identified on the en face image (poorly delineated or potentially fragmented FAZ) were excluded from the analysis.

**Figure 2.**
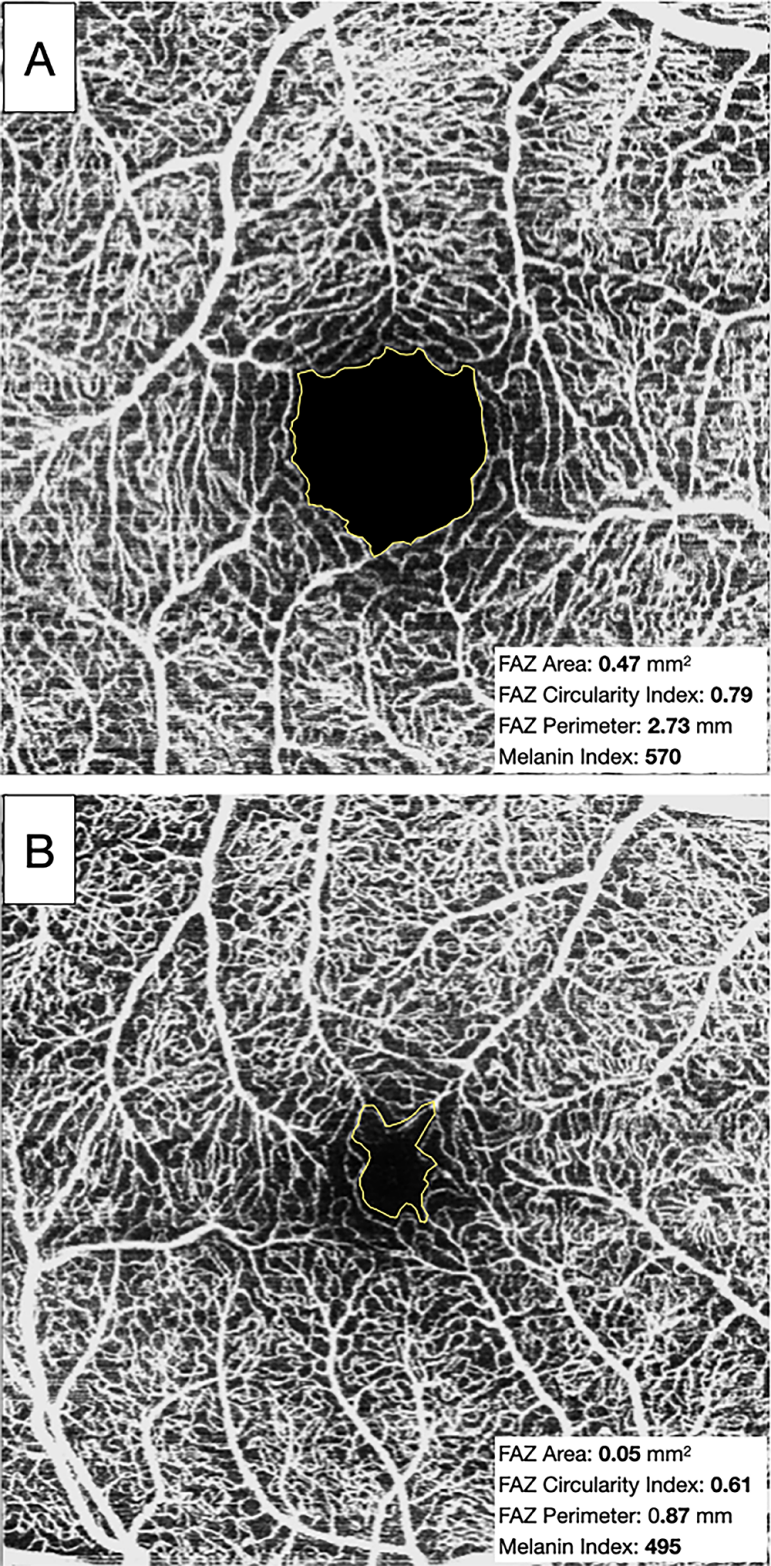
Analysis of the FAZ using ImageJ. The FAZ boundary was manually delineated. (**A**) Larger and more linear FAZ. (**B**) Smaller and more irregular FAZ. The FAZ area, FAZ circularity index, FAZ perimeter, and melanin index values for both OCTA scans are indicated alongside each respective image.

After setting the measurement scale based on the known image resolution (320 pixel/mm), the FAZ boundary was manually delineated by two independent graders (VF, AM) using the freehand selection tools. Measurements were obtained using the “Measure” command with the “Shape Descriptors” option enabled (area, perimeter, circularity). Circularity, which quantifies irregularities in shape border, ranges from 0 to 1 and was automatically calculated by ImageJ as indicated in other studies (Fig. 2).^32^^,^^33^ To account for interindividual differences in ocular magnification, FAZ area measurements were corrected for AL-dependent lateral magnification prior to statistical analysis, using the method described by Grieshop et al.^32^ Manual imaging analyses were performed by two trained retinal specialists (VF, MC).

### Melanin Assessment

Cutaneous melanin content was measured using a handheld colorimeter (SkinColorCatch; Delphin Technology, Bergisch Gladbach, Germany), which employs tristimulus colorimetry and narrow-band spectrophotometry.^34^ The device calculates a melanin index by analyzing light absorption and reflection across specific wavelengths: green (568 nm) and red (660 nm) for hemoglobin and red plus near-infrared (880 nm) for melanin. To minimize confounding from sun exposure and local skin conditions, melanin measurements were obtained at standardized, non–sun-exposed body sites (inner upper arm, posterior neck, lateral iliac crest), following protocols previously used for assessing constitutive pigmentation in dermatologic research.^35^^–^^37^

Each site was measured three times, and the average melanin value across all sites was used for analysis (see Supplementary Fig. S1). All measurements were performed in the same room with no daylight and under controlled ambient conditions, and the instrument was correctly calibrated before every measurement.^37^

### Statistical Analysis

The distribution of quantitative variables was assessed using both quantile–quantile (Q-Q) plots and the Shapiro–Wilk test. Data are reported as mean ± SD, median (interquartile range [IQR]), or frequency (percentage) as appropriate. To quantify the level of agreement between the readers, we utilized Cohen's κ test and intraclass correlation coefficients (ICCs) when appropriate. Initial associations between the FAZ parameters (area, perimeter, circularity index) and the melanin index were evaluated using Pearson's correlation. Subsequently, linear regression models were applied to confirm associations, adjusting for key covariates. Specifically, a univariable model first examined the relationship between the FAZ area and the melanin index. This was followed by a multivariable linear regression, including AL, age, and sex. To evaluate predictors of inner retinal layer persistence, a generalized linear model with a binomial distribution was implemented. Predictor variables included the melanin index, AL, and age. Model performance and goodness-of-fit were assessed to ensure robustness. All statistical analyses were conducted using R 4.2.1 (R Foundation for Statistical Computing, Vienna, Austria). A significance threshold of *P* < 0.05 was applied, and all tests were two tailed.

## Results

A total of 154 eyes from 154 participants (106 females, 68.8%) were included in this study. The median age was 32 years (IQR, 26–51 years). Among these, 21 individuals (13.6%) exhibited persistent foveal inner retinal layers on OCT. Table 1 summarizes the demographic and ocular characteristics of the study population.

**Table 1.** Demographics and Ocular Characteristics (*N* = 154)

Characteristic	Value
Age (y), median (range)	32 (26–51)
Female gender, *n* (%)	106 (68.8)
Laterality, right eye, *n* (%)	84 (54.5)
AL (mm), mean ± SD	24.05 ± 1.03
FAZ area (mm^2^), mean ± SD	0.28 ± 0.11
FAZ circularity index, mean ± SD	0.74 ± 0.11
FAZ perimetry (mm), mean ± SD	2.07 ± 5,4
CMT (µm), mean ± SD	274 ± 21
CFT (µm), mean ± SD	227 ± 19
Persistence of inner retinal layers (yes), *n* (%)	21 (13.6)

### Agreement Between Readers

The ICC between graders for FAZ area, perimeter, and circularity index was found to be >0.90, which indicates good agreement. For the qualitative assessment of the persistence of foveal inner retinal layers, the Cohen's κ was found to be 0.97 (95% confidence interval [CI], 0.93–1.00).

### Melanin Index and FAZ Metrics

Skin melanin levels, expressed as the melanin index, showed considerable variability across participants (range, 495–695; mean ± SD = 573.6 ± 34). A positive and statistically significant correlation was observed between the melanin index and FAZ area (*r* = 0.397, *P* < 0.001), as well as between the melanin index and FAZ perimeter (*r* = 0.415, *P* < 0.001). As expected, FAZ area and perimeter were highly interrelated (*r* = 0.91, *P* < 0.001). Given the wider use of FAZ area in clinical practice, subsequent statistical models used this parameter as the primary outcome.

**Figure 3.**
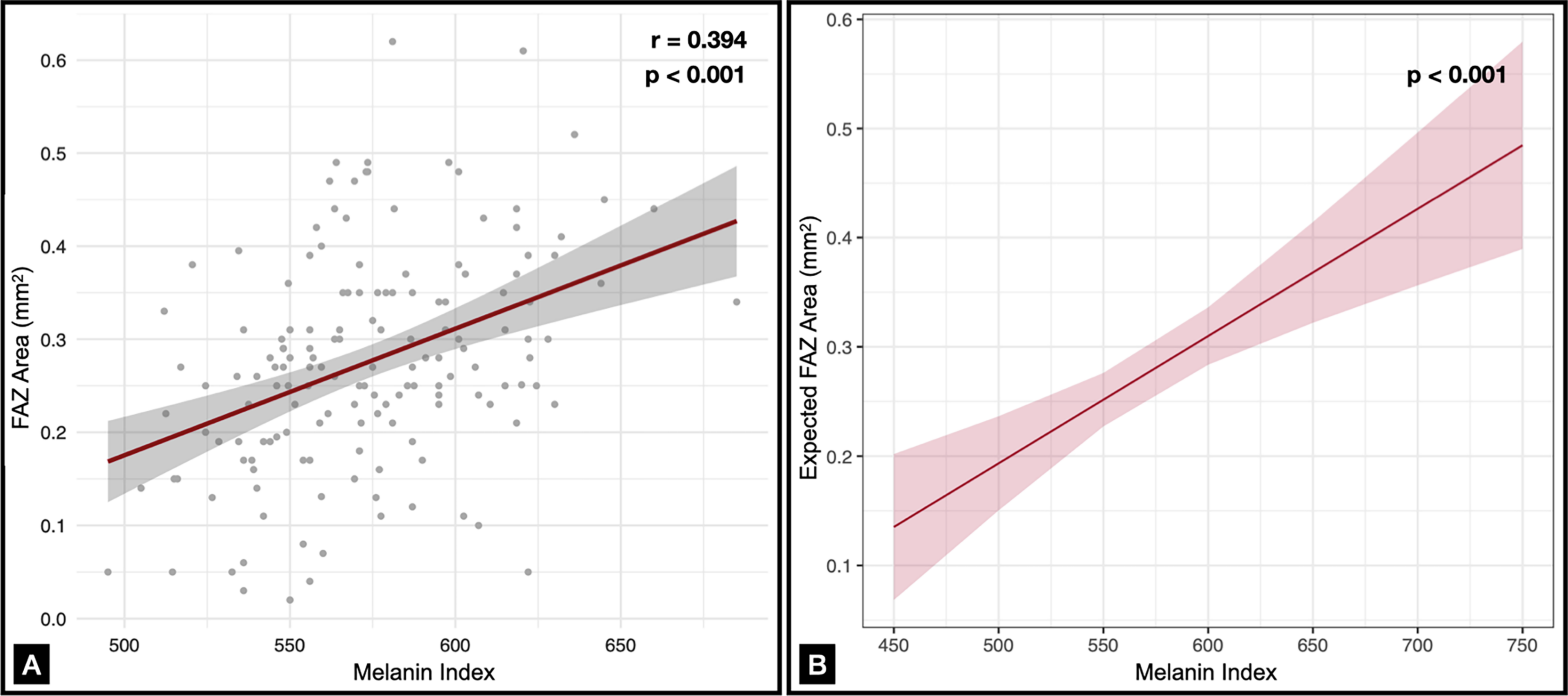
(**A**) Significant positive correlation between FAZ and the melanin index (*r* = 0.394, *P* < 0.001). (**B**) Results of a predictive multivariable linear regression model, which estimates changes in FAZ as a function of variations in the melanin index, after adjusting for AL, age, and sex. The analysis indicates that, when accounting for all variables, each 100-unit increase in the melanin index (M) corresponds to an increase of 0.12 mm^2^ in FAZ area (*P* < 0.001).

In the multivariable linear regression model including axial length, age, and sex as predictors, the melanin index remained a significant positive predictor of FAZ area. In particular, each 100-unit increase in the melanin index was associated with a 0.12-mm^2^ increase in FAZ area after adjusting for AL, age, and sex (β = 0.001, *P* < 0.001, *R*^2^ = 0.12). (Table 2; Fig. 3). Neither age nor sex and axial length were significantly associated with FAZ area. No significant associations were found between the melanin index or any other variables and the FAZ circularity index.

**Table 2.** Analysis of Factors Predicting FAZ Area

Predictors	Estimate	SE	*t*	*P*
Intercept	0.4135	0.2530	−1.634	0.104
Melanin index	0.0012	0.0002	4.454	<0.001
AL	−0.0007	0.0084	−0.033	0.974
Age	0.0007	0.0006	1.109	0.269
Male gender	−0.0277	0.0190	−1.458	0.147

### Melanin Index and Retinal Thicknesses

Pearson's correlation analyses showed a strong inverse relationship between FAZ area and both CMT (*r* = −0.66) and CFT (*r* = −0.54; both *P* < 0.001). Further regression analysis identified the melanin index and age as significant predictors of CFT. Specifically, the melanin index was negatively associated with CFT (β = −0.11, *P* = 0.02), indicating that higher melanin levels are linked with a thinner fovea, whereas age was positively associated with CFT (β = 0.23, *P* = 0.03), indicating increased foveal thickness with age. AL was not significantly associated with CFT in this model (*P* = 0.28). By contrast, no significant predictor was identified for CMT in the multivariable model.

### Melanin Index and Persistence of Foveal Inner Retinal Layers

In the generalized linear model evaluating the association of the melanin index, AL, and age with persistence of the foveal inner retinal layers, only the melanin index was a significant predictor, with higher melanin values associated with lower odds of persistence of foveal inner retinal layers (OR = 0.98; 95% CI, 0.97–1.00; *P* = 0.04). Neither AL nor age showed significant associations (both *P* > 0.05). The model, however, demonstrated modest explanatory power (Tjur's *R*^2^ = 0.058), suggesting that additional factors may contribute to the persistence of foveal inner retinal layers (Table 3).

**Table 3.** Logistic Regression Results Predicting Persistence of Inner Retinal Layers

Predictors	Odds Ratio	95% CI	*P*
Intercept	13,142.62	0.01–36,378.18	0.196
Melanin index	0.98	0.97–1.00	0.044
AL	0.97	0.60–1.51	0.884
Age	0.97	0.93–1.01	0.194

## Discussion

In this study, we investigated the relationships between cutaneous melanin levels and various foveal parameters, including FAZ metrics, in a cohort of healthy White individuals. We identified a significative positive correlation between the melanin index and FAZ area, suggesting that melanin may play a developmental and regulatory role in shaping the FAZ and foveal morphology, even within a single ethnic group. These findings indicate that pigmentation may have to be taken into account when using FAZ as a biomarker of retinal perfusion in both ocular and systemic diseases, as adjustment for pigmentation may enhance the accuracy of data interpretation.

Our work stems from observations in albinism, a group of genetic disorders characterized by impaired melanin synthesis and associated foveal development anomalies in almost all affected patients. These include foveal hypoplasia and absent or underdeveloped FAZ, reinforcing the idea that melanin plays a critical role in foveal and FAZ morphogenesis.^21^ Previous studies have demonstrated inter-ethnic differences in foveal architecture, suggesting that pigmentation may be linked to specific structural features of the fovea.^38^ For example, Wagner-Schuman et al.^39^ reported that African-American individuals had significantly deeper and wider foveal pits than White subjects. Similarly, Nolan et al.^40^ observed that White participants had a significantly narrower foveal width, approximately 100 µm less, than non-White individuals. Our findings also indicate that physiological variations in melanin levels among healthy White individuals could have a role in shaping the foveal structure.

Moreover, we not only evaluated structural parameters such as CFT and CMT, but we also evaluated vascular parameters derived from OCTA, finding a correlation between melanin and FAZ area. The FAZ is increasingly recognized as a meaningful biomarker for detecting and monitoring various retinal and systemic conditions. In diabetic retinopathy and age-related macular degeneration, abnormalities in FAZ size or shape have been correlated with disease severity and progression.^12^^,^^13^^,^^41^ Consequently, FAZ metrics obtained through OCTA are becoming valuable tools to complement conventional clinical evaluations and refine treatment strategies for retinal diseases. As such, understanding physiological determinants of FAZ variability, such as melanin, is essential to refining the interpretability and clinical utility of this biomarker.

When considering retinal embryology, multiple studies suggest that FAZ formation precedes and guides foveal development.^21^^,^^24^^,^^42^^,^^43^ A key histological study by Provis^44^ examined infant retinas from postmortem donors with no retinal pathology. The findings, consistent with previous non–human primate studies,^45^^,^^46^ showed that retinal vascularization begins around the 14th week of gestation at the optic disc, spreading radially toward the developing fovea. By approximately 25 to 26 weeks, blood vessels reach the nasal margin of the future foveal pit, and, by 37 weeks, a capillary meshwork encircles the developing pit. This network defines an early FAZ, about 150 to 170 µm in diameter, within the rod-free zone. Importantly, Provis^44^ found no histological evidence of blood vessels invading and subsequently regressing from the FAZ center, as previously suggested. Instead, the FAZ observed at birth (∼150–170 µm in diameter) gradually expands to adult dimensions during the first year of life, in parallel with the widening and deepening of the foveal pit. Factors believed to regulate this process include PEDF, natriuretic peptide precursor B, collagen type IV alpha 2, and ephrin A6. Among these, PEDF plays a central role. This anti-angiogenic factor is expressed by the RPE and is regulated through melanin-related pathways.^42^^,^^43^^,^^47^ In brief, melanin synthesis begins with tyrosinase, which converts l-tyrosine to both l-DOPA and dopaquinone. l-DOPA then acts as a ligand for the RPE-localized receptor GPR143, which, when activated, promotes PEDF expression. PEDF, in turn, counterbalances VEGF activity, helping to maintain avascular regions such as the FAZ.^47^

In our study, we observed that for every 100-unit increase in the melanin index the FAZ expanded by 0.12 mm^2^, supporting the idea that higher melanin levels may enhance PEDF expression and anti-angiogenic activity, ultimately resulting in a larger FAZ. Interestingly, in our cohort, neither sex nor age significantly affected FAZ area. This contrasts with other studies that identified a linear increase in FAZ with age, possibly due to age-related capillary loss.^11^^,^^48^ An explanation for this difference could be that our participants were relatively young (18–55 years), and the relatively narrow age window may have reduced the impact of age-related vascular changes.^49^

We also found that lower melanin levels were associated with the persistence of foveal inner retinal layers over the ONL in the foveal pit, a hallmark of grade 1 foveal hypoplasia.^29^^,^^30^ Our findings suggest that melanin may be important not only for FAZ formation but also for proper foveal pit remodeling. Even in individuals with normal cone specification and preserved visual acuity, lower melanin may predispose to subtle foveal hypoplasia. Notably, individuals with persistent foveal inner retinal layers exhibited a smaller FAZ and greater central foveal and macular thickness, supporting previous findings that smaller FAZ size correlates with increased foveal thickness and mild foveal hypoplasia.^38^ However, additional unmeasured factors are also likely to contribute to the persistence of foveal inner retinal layers such as genetic background or developmental variability. Indeed, detailed gestational and perinatal data that could have had a role in foveal morphogenesis were not available for the included subjects.^50^^–^^53^ Moreover, early interactions between RPE and neuroblasts may also play a role, consistent with experimental evidence from albino models showing compromised RPE integrity and disturbed foveal morphogenesis.^54^^,^^55^ Age was found to be positively associated with CFT, with older participants exhibiting greater central retinal thickness. This observation appears to contrast with findings from other studies, which report a progressive thinning of the retina with advancing age. However, it is important to highlight once again that the subjects included in our study were between 18 and 55 years of age. Thus, although CFT does increase with age, this finding is limited to a cohort of younger individuals and does not account for older age groups. Supporting this, von Hanno et al.,^56^ in a cohort study involving more than 4000 participants, reported that CFT progressively increases with age, peaking at approximately 60 years old, after which it begins to decline.

We acknowledge that there are some limitations that must be considered when interpretating our findings. A primary limitation of our study is that cutaneous melanin measurement may not provide a precise quantitative estimate of melanin within ocular tissues. We therefore used the melanin index as a practical proxy of overall pigmentation background, based on evidence of partial biological overlap between skin and ocular pigmentation.^57^ Both skin melanocytes and RPE cells share the canonical melanogenesis pathway, including key enzymes—tyrosinase (TYR), tyrosinase-related protein 1 (TYRP1), and dopachrome tautomerase (DCT)—and overlapping pigmentation genes (e.g., *TYR*, *OCA2*–*HERC2*, *SLC45A2*, *MC1R*) that contribute to inter-individual variation.^58^^–^^61^ In addition, TYR loss-of-function variants can reduce pigmentation in multiple compartments, including skin and the RPE, indicating that some melanogenesis components act across tissues; however, pigmentation is regulated in a tissue-specific manner, and these compartments are not equivalent.^62^ Therefore, cutaneous pigmentation does not precisely reflect ocular melanin content, but it may still provide a general indication of an individual's pigmentation background and, to some extent, of ocular melanin expression. Related to this, another limitation of our study is the lack of genetic data. Previous work has shown that hypomorphic TYR variants are associated with differences in FAZ area.^63^ Future research should be directed toward integrating genotypic data with phenotypic pigmentation measurements to more clearly delineate the determinants of FAZ area. A third limitation of our study is that the SkinColorCatch colorimeter estimates the melanin index based on specific wavelengths of light absorption and reflection. Consequently, external factors such as skin hydration, recent sun exposure, or temperature may influence readings and potentially introduce variability that does not necessarily reflect true baseline melanin levels. In addition, although our results support melanin as a significant contributor to FAZ area and mild foveal hypoplasia, a considerable portion of the observed variability remains unexplained, and the absence of genetic ancestry information means that self-reported ethnicity cannot account for potential ancestral admixture.

Additional factors, such as genetic background, environmental exposures, or ocular parameters, influence FAZ morphology and warrant further investigation. For example, previous evidence indicates that variability in FAZ and foveal pit structure is closely linked to photoreceptor packing and RPE organization, further supporting the role of multiple, interconnected developmental pathways in shaping foveal specialization.^64^

## Conclusions

Our findings support the hypothesis that melanin contributes to the developmental and regulatory processes involved in foveal morphogenesis, highlighting the importance of pigment-related pathways in shaping normal retinal architecture. We propose that variations in skin pigmentation, often overlooked in clinical and research settings, should be taken into account alongside ethnicity, AL, and comorbidities when interpreting FAZ metrics as biomarkers in common retinal diseases. We believe that this study expands current knowledge on the factors influencing FAZ, although additional aspects remain to be investigated. For example, future research could aim to correlate cutaneous pigmentation, FAZ morphology, and iris pigmentation to further clarify how pigmentation across different ocular compartments relates to foveal features. In addition, future studies could examine whether cutaneous melanin levels and FAZ metrics correlate with fundus characteristics measured by near-infrared autofluorescence, given its ability to capture cumulative autofluorescence signals related to melanin in RPE cells.

## Supplementary Material

Supplement 1
