# Supplementary material for: Skin Melanin Content and the Foveal Avascular Zone Correlation in a Healthy White Population
Source: Invest Ophthalmol Vis Sci. 2026 Mar 18;67(3):43. doi: 10.1167/iovs.67.3.43 (PMC13007563; doi:10.1167/iovs.67.3.43)
Supplement: Supplement 1 [file iovs-67-3-43_s001.pdf]

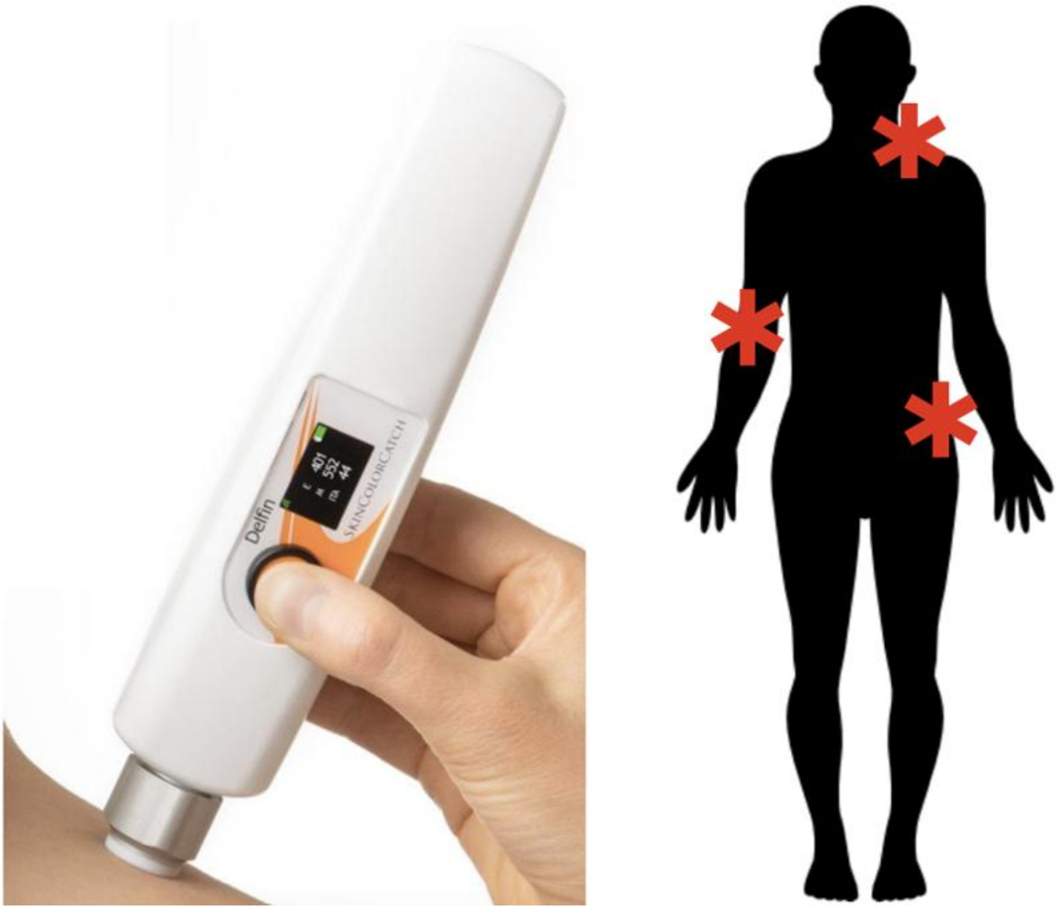

Supplementary Figure S1

The instrument shown in (A) is the SkinColorCatch device by Delfin Technologies: it is portable, battery operated and non-invasive. The red asterisks in (B) indicate the sites where measurements were taken to obtain the melanin index.
